# A text message intervention for quitting cigarette smoking among young adults experiencing homelessness: study protocol for a pilot randomized controlled trial

**DOI:** 10.1186/s13722-020-00187-6

**Published:** 2020-02-19

**Authors:** Joan S. Tucker, Eric R. Pedersen, Sebastian Linnemayr, William G. Shadel, Maria DeYoreo, Rushil Zutshi

**Affiliations:** 1 RAND Corporation, 1776 Main Street, PO Box 2138, Santa Monica, CA 90407-2138 USA; 2 RAND Corporation, 4570 Fifth Ave, Ste. #600, Pittsburgh, PA 15213 USA

**Keywords:** Cigarettes, Nicotine, Addiction, Intervention, Text message, Homeless, Young adults

## Abstract

**Background:**

Cigarette smoking is much more prevalent among young people experiencing homelessness than in the general population of adolescents and young adults. Although many young homeless smokers are motivated to quit, there are no empirically-evaluated smoking cessation programs for this population. It is important that any such program address the factors known to be associated with quitting-related outcomes among homeless young people, to provide ongoing support in a way that accommodates the mobility of this population, and does not rely on scarce service provider resources for its delivery. The objective of this project is to develop and pilot test a text messaging-based intervention (TMI), as an adjunct to brief cessation counseling and provision of nicotine patches, to help homeless young people who want to quit smoking.

**Methods/design:**

This pilot study will utilize a cluster cross-over randomized controlled design with up to 80 current smokers who desire to quit and are recruited from three drop-in centers serving young people experiencing homelessness in the Los Angeles area. All participants will be provided with a minimum standard of care: a 30-min group-based smoking cessation counseling session and free nicotine replacement. Half of these smokers will then also receive the TMI, as an adjunct to this standard care, which will provide 6 weeks of ongoing support for quitting. This support includes continued and more intensive education regarding nicotine dependence, quitting smoking, and relapse; does not require additional agency resources; can be available “on demand” to users; and includes features to personalize the quitting experience. This study will investigate whether receiving the TMI adjunct to standard smoking cessation care results in greater reductions in cigarette smoking compared to standard care alone over a 3-month period.

**Discussion:**

This study has the potential to address an important gap in the clinical research literature on cigarette smoking cessation and provide empirical support for using a TMI to provide ongoing assistance and support for quitting among young smokers experiencing homelessness.

*Trial registration* ClinicalTrials.gov Identifier NCT03874585. Registered March 14, 2019, https://clinicaltrials.gov/ct2/show/record/NCT03874585.

## Background

The goal of this project is to conduct a pilot evaluation of a text messaging-based smoking cessation program for young people experiencing homelessness. We are specifically interested in the “unaccompanied homeless youth” population, which is typically defined as individuals up to age 25 that are not currently living with or getting significant financial support from a parent or guardian, and who also have spent the previous night in a homeless setting (e.g., friend’s couch, street, homeless shelter) because of no place else to go [1–3]. The annual point-in-time U.S. homeless counts show an increasing number of unaccompanied young people experiencing homelessness, with the most recent count finding that 36,361 such young people are homeless on any given night [4]. In addition to living in poverty and being exposed to dangerous conditions inherent to street living, young people experiencing homelessness report high rates of cigarette smoking that put them at increased risk for the significant negative health effects associated with tobacco use [5].

## Cigarette smoking is prevalent among young people experiencing homelessness

Studies of unaccompanied homeless youth, which have tended to include both adolescents and emerging adults, have found that approximately 70% are current (past 30 day) cigarette smokers [6–8]. Further, 71% to 95% of these young smokers experiencing homelessness report smoking daily and 47% to 65% report smoking a half of a pack or more per day [8, 9]. These rates are substantially higher than in the general population of adolescents and young adults [10]. Further, research on homeless adolescents and young adults has found that most report one or more particularly high-risk smoking behaviors such as smoking shared cigarettes (96%), smoking discarded butts (71%) and filters (46%), and blocking filter vents (39%) [11]. Nearly half of young homeless cigarette smokers report rolling their own cigarettes, which may be filled with used tobacco obtained from discarded butts [12]. These practices may heighten their exposure to toxins and susceptibility to highly infectious diseases such as influenza, infectious hepatitis A, and tuberculosis [13, 14]. The health of homeless young people is adversely affected by unsafe living environments, nutritional deficiencies, mental health problems, problematic substance use, and insufficient access to health services [8, 15–18]. Adding tobacco use to the mix promises to further impair their already compromised health functioning [19].

## Young people experiencing homelessness are interested in formal smoking cessation services

The vulnerable population of young people experiencing homelessness has been largely overlooked in efforts to reduce smoking. Very few studies have examined the correlates of smoking among homeless adolescents and young adults [8, 20, 21], and there are currently no empirically-evaluated smoking cessation programs that specifically address the needs of this population. Yet, young smokers experiencing homelessness are motivated to quit smoking. For example, in a sample of nearly 300 homeless adolescents and young adults recruited from street venues, we found that almost half (43%) were motivated to quit in the next 30 days, and 76% of those who were motivated to quit were interested in using a nicotine replacement product and/or smoking cessation counseling to help them quit [9, 22]. Our work has found that homeless young people who are interested in quitting enjoy the camaraderie and peer support that group-based programs offer [23]. Thus, an initial group-based smoking cessation counseling session could provide a key opportunity to strengthen their motivation to quit and provide nicotine replacement medication. Yet, ongoing support is likely needed to help keep these young people engaged and commited to quitting smoking.

## Service providers consider smoking cessation a priority, yet have limited resources

Service providers are interested in helping homeless young people quit smoking, but few programs are currently offered [24, 25]. Our interviews with service providers found that nearly all (95%) were interested in offering cessation programming, but only one had an ongoing policy for helping their smoking clients (referrals to the California Smokers’ Helpline). The most commonly cited barrier to implementing a formal cessation program on site was lack of resources and staff training; thus, providers indicated a strong preference for cessation programs that required fewer resources (e.g., single session rather than multi-session treatment) but were also intensive enough to keep the client engaged and supported. A mobile health (mHealth)-based tool that provides ongoing support for quitting is likely a more sustainable approach than one that would rely on additional service provider resources.

## Text messaging interventions (TMIs) may be a promising approach

A recent Cochrane meta-analysis of randomized controlled studies that had follow-ups of 6 months or longer found that TMIs increased quit rates by 50–60%, both when the TMI was compared to minimal support and when it was tested as an adjunct to other forms of cessation support [26]. While these studies were mostly based on general adult samples, with only four studies specifically targeting young adults, using a TMI for smoking cessation may be a promising approach for young smokers experiencing homelessness. Nearly universal cell phone ownership among young people experiencing homelessness [27] holds great potential for offering ongoing support for behavior change as an adjunct to face-to-face services. A recent study using text messaging for daily data collection among homeless young people found it to be both acceptable and feasible; for example, individuals reported that receiving the texts made them feel that someone cared about them and encouraged them to self-reflect on their life [28]. There is growing appreciation that cell phones are not a luxury for those experiencing homelessness, but rather a necessity in terms of helping them maintain social and service contacts [29, 30]. Indeed, a number of recent initiatives have focused on increasing homeless individuals’ access to mobile technology for these purposes [31].

A tailored approach to smoking cessation which combines a single group counseling session and nicotine replacement with a TMI that is tailored to the circumstances of young smokers experiencing homelessness and the key factors that are known to be particularly relevant to their smoking cessation [9] could help circumvent the formidable barriers to quitting in this population. Such a TMI adjunct to a group-based treatment can provide ongoing information and support for quitting. This is especially important in service environments where both resources and the window for intervening with these young people are limited. Of course, there is much to learn about the feasibility of using TMIs for young people experiencing homelessness. For example, their cell phones may be more prone to loss or theft, or limitations in terms of data plans and functionality (ability to access websites, staying charged). These types of issues will be examined in this pilot study.

## The present study

The considerable evidence for the efficacy of TMIs to support quitting behavior suggests that this approach should be a public health priority. Further, innovative strategies are needed that can capitalize on existing motivation and initiative to quit smoking among young people experiencing homelessness, and provide the necessary resources and ongoing support to help them achieve their cessation goals. This study protocol describes our work to develop and pilot test a TMI designed to help young people experiencing homelessness to quit smoking.

## Methods/design

### Overview

This pilot study involves a cluster cross-over randomized controlled design [32–34] with up to 80 current smokers who desire to quit and who are recruited from three drop-in centers serving young people experiencing homelessness in the Los Angeles area. Two of the drop-in centers are approximately a two mile drive from each other, with the third drop-in center being approximately a 10–15 mile drive from the others. The unit of analysis will be the individual, but individuals will be assigned to groups (standard care alone vs. TMI adjunct) based on the drop-in center where they are seeking services. All participants will receive standard care: a 30-min group-based smoking cessation counseling session and free nicotine replacement. Half of these smokers will then also receive the TMI, as an adjunct to this standard care, which will provide 6 weeks of ongoing text messaging support for quitting. This ongoing support includes continued and more intensive education regarding nicotine dependence, quitting smoking, and relapse; does not require additional agency resources; can be available “on demand” to the user; and includes features to personalize the quitting experience. During the field period, each drop-in center will alternate in delivering these two treatments, offering one group-based smoking cessation counseling session every other month (to reduce possible contamination between conditions), with the type of treatment delivered first at each drop-in center being randomly determined. This pilot study will investigate whether receiving the TMI adjunct to standard smoking cessation care results in greater reductions in cigarette smoking compared to standard care alone over a 3-month period.

### Participants

We will recruit participants using the following eligibility criteria: (a) between the ages of 18–25; (b) unaccompanied homeless (defined as not currently living with or getting significant support from a parent or guardian, and having spent the previous night in a shelter or other homeless setting because of no place else to go); (c) smoked at least 5 cigarettes per day on at least 20 days in the past month; (d) ready to set a quit date in the next 30 days; and (e) have a cell phone that can receive text messages. Individuals will be ineligible if they: (a) are currently pregnant or breastfeeding, or planning to become pregnant or breastfeed, in the next 6 months (females only); (b) have a medical condition (based on participant self-report) which would prevent using nicotine replacement (e.g., allergy to adhesives, heart disease); (c) have used pharmacotherapy to reduce or stop smoking in the past 30 days; and (d) are currently receiving other smoking cessation services. We focus specifically on young adults in this study for four reasons. First, the vast majority of the unaccompanied homeless youth population is between 18 and 25 years old [4]. Second, this is the age range served by the participating agencies, so we will not have to refuse treatment to anyone on the basis of age. Third, a wider age range among participants may adversely affect group cohesion and dynamics for the group-based counseling session. Finally, important developmental differences between adolescents and emerging adults would likely require tailoring of the program curriculum, which is beyond the scope of this pilot study.

### Recruitment and follow-up procedures

We will recruit individuals for the program by posting flyers and making announcements about the program at the drop-in centers. This is an approach that we have successfully used in a number of previous studies over the past 15 years to recruit young people from shelters and drop-in centers [2, 34–37]. Individuals who are interested will be asked to answer a few questions to determine eligibility (see above). Verbal consent will be used for the screener and eligible individuals will then be asked to provide written informed consent. Research staff will read the consent form aloud to the participant, who will follow along from their own copy, and answer any questions that the participant might have before signing the form. A Certificate of Confidentiality has been obtained for this project to protect collected data from subpoena. Given that we are offering a monetary incentive, some participants may be motivated to participate in the study more than once. We will employ a combination of procedures that we have successfully used in past studies to minimize the likelihood of having repeaters in the sample [e.g., not screening individuals identified by field staff as known repeaters; using information participants provide on the tracking/locator form and surveys (e.g., background information) to weed out repeaters from the database].

**Fig. 1:**
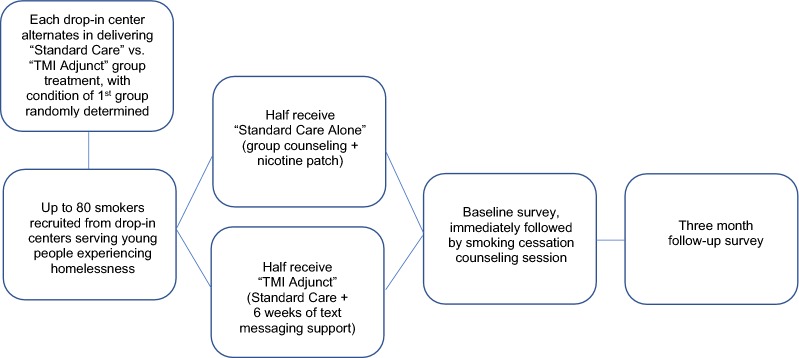
Randomized controlled trial study flow

**Fig. 2:**
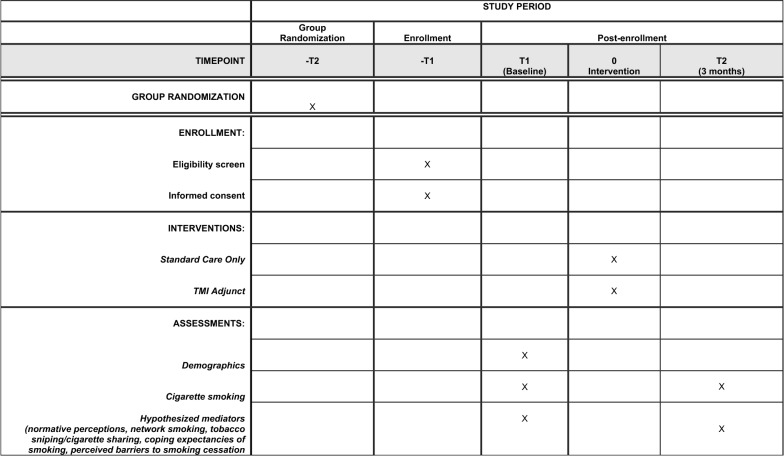
SPIRIT flow diagram of the RCT schedule of enrollment, interventions, and assessments

Enrolled participants will complete their baseline survey while waiting for the 30-min group-based smoking cessation counseling session to start. After the counseling session, participants randomized to the TMI condition will begin receiving 6 weeks of text messages to their personal cell phone. Three months after the baseline survey, all participants will receive a follow-up survey. We expect a very high retention rate at 3-month follow-up based on the 91% retention rate we achieved at 3-month follow-up in a recent evaluation of a substance use and sexual risk reduction program involving 200 homeless young people conducted at some of the same study sites participating in this project [38]. We will use a number of innovative retention procedures that have helped us limit attrition with this transient population, such as collecting tracking information on their hangouts, places used for sleeping outdoors, and social networking sites; see our previous work for a detailed description of these procedures [39]. Figure 1 shows participant flow through the study, and Fig. 2 contains a SPIRIT (Standard Protocol Items: Recommendations for Interventional Trials) flow diagram of the RCT schedule of enrollment, interventions, and assessments.

### Intervention setting

Recruitment and group counseling sessions will be conducted within several drop-in centers in Los Angeles County. Drop-in centers are designed to be a low barrier, “come as you are” point of service entry for young people experiencing homelessness. Drop-in centers provide a temporary respite from the streets and offer both basic (e.g., food, showers) and higher-level services (e.g., case management; employment, education, and health programs). Drop-in centers tend to be preferred by young people over other service settings (e.g., shelters) which have more rules and regulations [40]. Thus, drop-in centers are an ideal setting to reach young people experiencing homelessness who may not seek services elsewhere.

### Description of the intervention

Our prior work with young homeless smokers identified several important factors that should inform the content of any smoking cessation program for this population [9]. We address each of these factors in both the group counseling session and the text messaging-based intervention.

#### Smoking is normative

The pervasiveness of smoking can pose a significant challenge to quitting for young people experiencing homelessness, and thus it is important to connect them with others who can provide support for quitting. Indeed, young smokers experiencing homelessness that we surveyed tended to prefer a group-based program that would provide ongoing support for quitting over one-on-one counseling [23]. Increasing exposure to smokers who want to quit smoking may result in smoking being perceived as less normative, which our work shows is related to greater motivation to quit among homeless young people [9]. This can be targeted in both the counseling session, where participants are exposed to peers who are also trying to quit smoking and encouraged to be sources of support for each other, as well as through the TMI via texts with statements about how most smokers actually desire to quit and many eventually are successful. The TMI also provides strategies for dealing with peers who smoke or who are not supportive of the participant’s efforts to quit smoking.

#### Cost and high-risk smoking practices

We have found that young smokers experiencing homelessness spend, on average, one-third of their monthly income on cigarettes [9]. Due to the high cost of smoking, most of these young smokers also engage in high-risk smoking practices (e.g., sniping discarded butts, puffing on others’ cigarettes) [11] which provide free tobacco, but pose additional health risks and are generally viewed by these young people as disgusting and unhealthy [23]. Our work suggests that highlighting both the cumulative costs of purchased tobacco, and the additional health risks posed by sniping, may increase motivation to quit smoking [9]. Thus, both the group counseling session and the TMI texts provide useful information about cost savings and dangers of high-risk use. For example, regarding costs, participants in the TMI condition are provided with a cost calculator that tells them how much they would save if they were to quit smoking. Once they have quit, at an interim time point of the TMI, they will be shown how much they have saved thus far due to quitting.

#### Smoking to reduce stress

Young homeless smokers typically view smoking as an effective way to cope with the stress they experience from being homeless, and perceive this stress as a major barrier to quitting [9]. As such, it is important that cessation programs for this population address myths about smoking and mental health (e.g., quitting smoking will take away a coping mechanism; they will experience increased mental health symptoms) and help these young smokers generate healthier alternatives to cope with stress. General information on coping is included in the counseling session. Then for TMI participants, texts address specific non-smoking coping strategies, and how nicotine can actually worsen anxiety symptoms. In addition, TMI participants are able to request stress-reduction strategies on demand by texting a keyword (“Mood”); the keyword generates a coping strategy to help participants when they are feeling particularly stressed or depressed.

#### Smoking cognitions

Motivation to quit is significantly stronger among homeless young people who are confident in their ability to quit and perceive fewer barriers to quitting [9]. This suggests that cessation programs for this population may be more effective to the extent that they can increase their general motivation to quit, as well as help them identify strategies for dealing with their cravings and personal triggers for smoking and offer concrete strategies for quitting. Both the counseling session and the text messages address these issues. In addition, TMI participants can text the keyword “Crave” to receive specific non-smoking coping strategies on demand throughout the quitting process.

The project team generated 174 text messages based on the factors just described, as well as consulting with the text messaging literature and reviewing text messages included in other public domain smoking cessation programs such as Text2Quit (https://text2quit.com; [41]) and SmokefreeTXT (https://smokefree.gov/smokefreetxt). The majority of these texts address one of the five main foci of the intervention, based on our prior work identifying factors associated with motivation to quit among young homeless smokers [9]: strategies for getting support for quitting; calculations for the amount of money saved by quitting; presentation of health and social benefits of quitting; strategies for dealing with cravings and negative moods; and tips for staying motivated (see Table 1). Other texts included reminders to use the nicotine patches they were given, periodic check-ins to see if they were still reading the texts (“We just want to know that you got this text. Please text back: YES”), and occasional “fun” content (e.g., encouraging or funny memes). In developing the pool of text messages, care was taken to ensure that the content reflected the unique circumstances of young people experiencing homelessness. Importantly, to make messages more effective we used recent insights from behavioral economics in the design of the messages, such as using gain-/loss- framing *(‘Increase your chances of success by starting to use the nicotine patches tomorrow morning’*), employing social norms *(‘You’re not alone in wanting to be smoke*-*free. Most people your age don’t smoke… and most people that do smoke want to quit.’*), appealing to participants’ self-identity *(‘Look in the mirror and tell yourself: I am a nonsmoker! Staying smoke*-*free can be easier if you think of yourself as a “nonsmoker” instead of an “ex*-*smoker.”’*), and providing progress information to keep the salience of the quitting behavior high *(‘You’ve worked really hard for almost 2 weeks to get where you are now. Don’t lose this energy!’*).

**Table 1:** Main TMI program foci and example texts

Main program foci	Example texts
Staying motivated	“Write a message of encouragement to your future self and send it as a reply to this text. We’ll send it back to you as a surprise over the next few days” “Say out loud ‘I’m a nonsmoker.’ It seems cheesy, but it can remind you of all the changes you’ve made and help you stay strong through the cravings”
Getting support from others	“Hanging out with smokers can make quitting even harder. If you can’t avoid smokers for the first few days, tell them you’re quitting and ask for their support!” “Check in with your friends and let them know how you’re doing with staying smoke-free. If you need support from them, ask for it!”
Dealing with cravings and negative moods	“Don’t lose the progress you’ve made. Ride through cravings by chewing gum, walking it off, or taking 10 deep breaths. And text CRAVE for more support anytime.” “If you’re feeling down, stressed, or anxious, check out this link for ideas of things you can do to boost your mood: [link to external website]”
Health and social benefits of quitting	“Sniping discarded butts might be free tobacco—but it can also make you sick from germs on the ground or from the person who smoked it first. Not worth it.” “Quitting can make you instantly more attractive—surveys show that most people prefer kissing someone who doesn’t smoke!”
Amount of money saved by quitting	“Quitting smoking can save you some big $$! If you’re curious about how much you can save by quitting, check out this link: [link to external website]” “Think about what you’ll do with all the money you save by not smoking… and what you might buy for yourself once you finish this program]”

### Group counseling session and nicotine replacement

Participants in both conditions (standard care alone and TMI adjunct) will receive a 30-min counseling session, up to an 8-week supply of nicotine patches, and handouts on quitting smoking and dealing with cravings. The counseling session will be delivered in a small group setting at each of the drop-in centers. As mentioned earlier, each drop-in center will offer one group smoking cessation counseling session every other month, alternating in delivering the “standard care alone” vs. “TMI adjunct” treatment (with the type of treatment delivered during the 1st session at each drop-in center randomly determined). The counseling session will follow the 5 A’s format (Ask; Advise; Assess; Assist; Arrange) recommended in the 2008 U.S. Department of Health and Human Services Clinical Practice Guidelines for treating tobacco use and dependence [42, 43]. The session will be similar to our previous clinical research with adult smokers [44, 45]. However, it will be informed by our formative work and incorporate the content particularly relevant to young people experiencing homelessness (e.g., economic costs associated with smoking; health risks associated with free tobacco obtained through high-risk smoking practices) and use language appropriate for the young adult population. Participants will be asked to set a quit date and given an initial 4-week supply of nicotine patches with instructions on its use that follow package insert guidelines and potential side effects (participants will be able to get an additional 4-week supply of nicotine replacement, if they are interested; thus, they will have access to up to 8 weeks of nicotine replacement, a standard recommended length of treatment. Dosing instructions are tailored based on daily smoking rate. Two trained facilitators will be present at each session, one to deliver the intervention and the other to assist with the baseline survey, distributing materials, answering questions, and helping set up the TMI for those assigned to receive it.

### TMI adjunct to standard of care condition

**Fig. 3:**
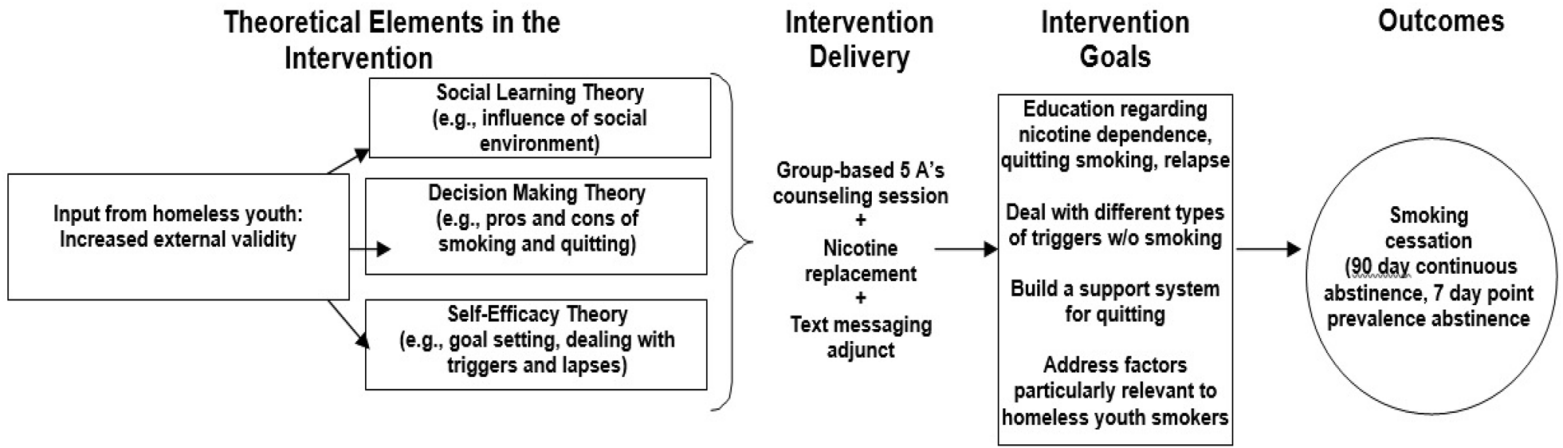
Conceptual model

At the end of the group counseling session, participants randomized to the TMI condition will be signed up to receive the text messaging-based intervention. As shown in Fig. 3, the intervention has a strong theoretical basis in Social Learning Theory (i.e. smoking is related to both modeling of others’ behavior and perceptions about the behavior of others [46, 47]); Decision Making Theory (i.e. decisions about engaging in smoking are often emotional and therefore problem-focused coping skills are needed [48, 49]); and (c) Self-Efficacy Theory (i.e. building confidence about quitting will increase the likelihood of a successful quit attempt [50]). Participants assigned to the TMI will receive automated text messages for 6 weeks, which is within the range of other TMIs for smoking cessation [51]. Text message frequency and content is tailored to whether it is a pre-quit day, quit day, early post-quit day (within 14 days of quitting), or late post-quit day (15 + days after quitting) [52], with frequency being highest early in the quitting process (i.e., on the quit and early-quit days) and then tapering down. See Table 2 for the TMI text flow and samples of text messages.

**Table 2:** TMI text message flow and sample texts

Timepoint of TMI	Number of texts per day	Sample texts
Pre-quit texts (Days 1 to 2)		
2 days before quit day	5 texts	“Think about when and why you were most tempted to smoke today. Write down a plan for how you’ll deal with these triggers on your quit day”
1 day before quit day	5 texts	“Why do you want to quit smoking, [name]? Text us the most important reason in 1 or 2 words. We’ll send it back to you when you need motivation!”
Quit day texts (Day 3)		
Quit day	7 texts	“Today is the day you’ve decided to quit—you can do it! Drinking lots of cold water today will help. We’ll text you throughout the day to support you.
Early post-quit texts (Days 4 to 16)		
1 day post-quit	6 texts	“Getting through the 1st day is tough, but you did it [name]! Celebrate by doing something nice for yourself today!”
2 days post-quit	6 texts	“Cravings are normal and they pass, whether you smoke or not. Ride them out by distracting yourself with a walk, listening to music, or visiting a friend”
3 days post-quit	6 texts	“Sniping discarded butts might be free tobacco—but it can also make you sick from germs on the ground or from the person who smoked it first. Not worth it”
4 days post-quit	5 texts	“If you’re feeling anxious, smoking isn’t the answer. Nicotine can make anxiety worse. Text MOOD anytime for tips on dealing with a bad mood without smoking.”
5 to 14 days post-quit	4 to 5 texts	“Letting friends know you’re quitting can make it easier for you and motivate them to quit too. Be proud of what you’ve achieved and let others know about it!” “The $$ you save by quitting can really add up! To see how much you’ve already saved, text back the typical # of cigs you smoked per day prior to quitting”
Later post-quit texts (Days 17 to 42)		
15 to 39 days post-quit	3 to 4 texts	“Using alcohol or marijuana can make it harder to resist cigarettes. If alcohol or weed are triggers for you, think about staying away from them for a bit”
2 days until end of program	4 texts	“Notice what’s gotten better since you changed your smoking. More energy? Easier breathing? Better tasting food? Enjoy these changes by staying smoke-free!”
1 day until end of program	4 texts	“If you’re wondering how much money you can save over the next year or even 5 years by staying smoke-free, check out this link…”
Last day of program	5 texts	“Write down the qualities and skills you used to stay smoke-free. Remind yourself of these things if you feel like giving into cravings in the future”

Consistent with most text messaging studies [51], we use strategies to make the TMI more personalized and interactive in order to enhance participant engagement. For example, some text messages incorporated the participant’s first name or nickname *(‘Keep up the great work [name]! Take a selfie when you’re not smoking and when you feel good. Then look at it when you feel a temptation to smoke’)*. Other text messages included probes that ask them to provide information *(‘Feeling stressed right now? Text back YES or NO’*) and then they would receive an automated response based on their answer. Similar to some other text messaging programs (e.g., SmokefreeTXT), participants could text CRAVE (“if you feel a craving or urge to smoke”), MOOD (“if you feel down, nervous, stressed, or bored”), or SLIP (“if you use and need some extra support”) anytime to automatically receive additional texts relevant to each of these three situations. The team developed an additional 72 text messages (24 each for “Mood,” “Crave,” and “Slip”) to be sent in response to these requests for additional support. In addition, some messages included hyperlinks to other information in the public domain that participants might find useful and interesting (e.g., calculating amount of money saved quitting, getting support from others for their quit attempt, finding no-cost smoke-free activities).

#### Formative work to develop the TMI adjunct: focus groups and usability testing

Existing TMIs for smoking cessation vary widely in the frequency of texting [51], and it was initially unclear what the optimal “dose” would be for young smokers experiencing homelessness. We also wanted to review the content of texts and make sure that receiving texts would be feasible for this population. Thus, we conducted a series of focus groups and elicited usability testing feedback with a small sample of young smokers recruited from the drop-in centers to inform decisions about the optimal content (e.g., what types of messages would likely be most effective in dealing with triggers) and wording (e.g., how to word the text message so that it motivates them to not smoke). Details of the work from this formative phase are published elsewhere [53]. Briefly, participants were 18–25 years old and recruited from the same drop-in centers as for the larger pilot. Three focus groups (N = 18) were conducted with smokers to refine the TMI content, and a separate sample of smokers (N = 8) provided feedback on the TMI after using it for 1 week. Survey data assessed the TMI’s acceptability and feasibility.

Participants generally rated the TMI as helpful and relevant, and nearly all had cell phone plans that included unlimited texting and were able to view TMI content with few difficulties. Potential logistic barriers to using the TMI over a 6-week period, such as losing their phone, were considered unlikely, while concerns about battery depletion, paying for data, or privacy of texts were also reported as of minimal concern. Qualitative feedback on strengths/limitations of the TMI in terms of content, tone, and delivery parameters was used to finalize the TMI. This feedback from participants helped to improve the TMI content for preparing to quit, staying motivated, getting support, dealing with cravings and negative moods, and stressing the health and cost benefits of quitting. It also helped us to modify wording of texts to be appropriate for the population, as well as include intermittent gifs, emojis, and memes to keep them engaged in the content.

### Analytic plan

Data will be collected from participants using self-administered paper–pencil surveys, with the survey response forms then scanned and checked for accuracy.

#### Missing Data and Attrition

We will assess missing data patterns at both time points, and will use a multiple imputation approach for these missing data where appropriate, using methods that are valid under missing at random (MAR) assumptions [54]. For example, we will create imputed values for all predictor variables. For outcome measures, different techniques will be used to ensure findings are robust to assumptions. If missingness is non-negligible (e.g., not less than 5%; [55]), then for those measures assessed only at follow-up, we will examine results using non-response weights, and will also conduct sensitivity analyses assuming that non-responders are still smokers as well as using multiple imputation techniques to impute the missing values. Our approach aims to account for study attrition while most efficiently utilizing all available data. For secondary outcomes assessed at both time points with non-negligible attrition, we will examine results using imputation models that include the baseline outcomes in the imputation model, and also using likelihood-based methods that are valid under MAR (e.g., the difference in differences model we describe later on).

#### Intent-to-treat sample and intervention non-compliance

Analyses will use the standard intent-to-treat (ITT) approach to examine the effect of the TMI Adjunct condition relative to the Standard Care Alone condition. Our ITT approach will analyze participants as belonging to the group they were randomized to, regardless of their compliance, because excluding those who do not use the TMI would bias results in favor of the TMI condition, increasing the probability of type I errors [56].

#### Preliminary analyses

Although participants will be randomly assigned (in groups of about 5–7 individuals) to either the TMI Adjunct or Standard Care Alone conditions, imbalance between the two groups on some of the baseline characteristics may occur. However, since assignment to treatment condition is random, any differences that arise are due to random chance [57, 58]. We will present balance tables of baseline data to confirm the two groups are similar. In all relevant analyses, we will control for variables that are expected to predict the outcome by including them as covariates in the models.

#### TMI feasibility and acceptability

Measures of TMI feasibility will include the percent of text messages that were delivered to the correct individual, in the correct sequence, and with appropriate responsiveness to each participant’s replies [59]. Measures of TMI acceptability will include percentages of participants who chose to stop receiving text messages, who continued to engage with the program (assessed by periodic prompts throughout the 6-week period to determine whether they were reading the messages), and who completed requested responses to the text messages [59]. At the 3-month follow-up, TMI participants will also complete survey items to obtain their feedback on text frequency, text content, ease of using the TMI, and whether they had trouble receiving texts in a timely fashion [60].

### Effects of the TMI on smoking behavior

The primary goal of analyses is to assess the promise of the TMI as an adjunct to standard brief cessation counseling and obtain preliminary estimates of intervention effect sizes for a larger trial of the TMI with homeless young people. Given the proposed small sample size, sophisticated modeling that makes adjustments for multiple covariates or potential non-response bias might not be possible; therefore, some analyses will be primarily descriptive.

Biochemically-verified 7-day point prevalence quit rates and self-reported continuous abstinence at 3-months following the baseline visit will be analyzed separately using logistic regression. Logistic regression will also permit modeling the effects of potentially important covariates (e.g., education, age, gender, nicotine dependence, smoking history). We will also compare average number of days smoked by comparing the 30 days immediately prior to the smoking cessation counseling session to the 30 days after the participant’s target quit date. We will fit a regression model to explain the difference in pre and post-intervention outcome by the randomization group indicator (TMI Adjunct vs. Standard Care Alone). We will also estimate a difference in differences (DID) approach for this outcome, including time (a dummy variable indicating pre- versus post-period), the intervention group indicator, and their interaction, and a random subject effect to properly account for the correlation of the repeated measures on each subject and produce efficient estimates of the intervention effect.

### Measures

Our primary and secondary smoking-related treatment outcomes are based on recommendations by the Society for Research on Nicotine and Tobacco [61]. The primary treatment outcome will be continuous abstinence, defined as no smoking from the target quit date through the 3-month follow-up. The secondary treatment outcome will be biochemically-verified 7-day point prevalence abstinence at the 3-month follow-up, with participants who report that they are abstinent having their status confirmed via expired air carbon monoxide (CO) using a CoVita Smokerlyzer ^®^ monitor and, in cases where the breath sample suggests that they are currently smoking, by saliva cotinine using a NicAlert cotinine test strip. Participants who report no smoking, but whose CO level > 5 ppm or cotinine level > 10 ng/mL, will be coded as smoking. We acknowledge that there is a possibility of carbon monoxide false positives related to smoking other substances such as marijuana. We will probe further about marijuana use if a participant reports no smoking but biochemical data suggest otherwise [62]. In addition, we will utilize the reliable and valid smoking time line follow-back (TLFB) procedure [63] to collect detailed information on smoking patterns in the 30 days prior to the cessation counseling session (assessed at baseline) and the 30 days after the participants’ target quit date (assessed at follow-up). This will be used to examine intervention effects on the average number of cigarettes smoked per day.

#### Effects of the TMI on other smoking-related outcomes

As a secondary focus, we will examine estimates of the intervention effect on proximal outcomes/mediators (i.e., factors associated with smoking and motivation to quit among young people experiencing homelessness [9] and areas of focus in the TMI) and assess trends. While the planned sample will not be large enough for detecting statistically significant mediation effects, it will allow us to produce estimates of the intervention effect on proximal outcomes/mediators and assess trends. We will adopt a similar modeling approach to the one just described, using a DID approach, since these variables will be measured at both baseline and follow-up.

#### Measures

*Normative perceptions* will be assessed by asking “Out of every 10 people your age without a regular place to stay, how many do you think smoke cigarettes?” (0 to 10; based on prior work [64]. *Smoking within the participant’s social network* will be assessed by asking about the proportion of friends who smoke (1 = *none* to 5 = *all*) [65]. *Frequency of tobacco sniping and cigarette sharing* in the past 30 days will be assessed with established items [11, 66]. *Coping expectancies of smoking* will be measured with the 4-item PROMIS Coping Expectancies of Smoking short form [67]. *Self*-*efficacy for quitting* will be assessed by a 14-item measure [68], and *perceived barriers to smoking cessation* will be assessed by a 13-item measure [69].

#### Power Considerations

A goal of this pilot study is to get an estimate of both the variability of the outcomes and of potential intervention effects so that we are better positioned to plan for the sample size for a future larger study [70]. To determine the necessary sample size for the larger study, we will determine what the clinically meaningful effects are for somewhat similar interventions from the literature. We will standardize such effects using the estimates of variability obtained with our pilot study data. For the pilot study, we plan to enroll up to 80 participants, with approximately 5–7 participants per group or cluster. We assume that 90% of them participate in the follow-up survey, based on our previous work [36], and assume a conservative interclass correlation (ICC) of 0.05. Under these assumptions, the effective sample size (ESS) at follow-up will be 36/(1 + 5*0.05) = 28.8 per condition. This provides 80% power to detect a moderate quit rate effect size (h = 0.72) for the difference between the two treatment conditions (alpha = 0.05).

## Discussion

### Limitations and alternative methods considered

The proposed intervention focuses exclusively on cigarette smoking and is not designed to address the use of electronic nicotine delivery systems (ENDS), poly-tobacco use, or the co-use of tobacco with cannabis or other substances. However, we will be able to explore intervention effects on these other forms of substance use in our analyses. It should also be noted that we are not providing cell phones to participants while they participate in this study; rather, individuals must have a working cell phone that can receive text messages in order to participate. We will be monitoring the number of individuals who screen ineligible for this reason, or have a phone that is lost or stops working during the intervention, as indicators of the feasibility of using TMIs with this population.

In terms of the research design, we initially considered a design in which individuals would be randomly assigned to condition within agency. However, we decided against this design given concerns on the part of the research team and drop-in center staff about implementation challenges (e.g., clients perceiving that they were being refused services that other clients were getting) and the strong potential for contamination across conditions. A group-randomized design was a much better option for this evaluation in that it addressed both of these concerns. We will maximize the comparability of the intervention and control groups by: (1) having each drop-in center serve as both intervention and control site on an alternating basis; and (2) using the same procedures at each drop-in center to identify and recruit participants for the study. In addition, once the data are collected we will conduct preliminary analyses to determine whether the intervention and control groups differ on baseline characteristics. This step is necessary despite randomization because the randomization is not occurring at the individual level. If we observe considerable differences in the intervention and control groups, we will control for these with the addition of covariates into the regression models. We may also consider developing analytic weights using propensity score methods to balance the characteristics of the groups. As mentioned earlier, we will also control for any within-site effects by including site as a fixed effect (dummy coded) within the models.

## Conclusion

Young people experiencing homelessness have alarmingly high rates of tobacco use, and are disproportionately affected by tobacco use, yet have been virtually ignored in efforts to reduce tobacco use among young people. This study will make a significant contribution to a very limited literature on cigarette smoking, and approaches to smoking cessation, in the highly vulnerable and underserved population of young people experiencing homelessness. It will be both the first to develop a TMI for smoking cessation, and the first to evaluate a smoking cessation program (of any kind), for young smokers experiencing homelessness. Further, although brief standard smoking cessation counseling and nicotine replacement are both well-established treatments, this study significantly extends research in this area by evaluating whether using a TMI for smoking cessation as an adjunct to brief counseling + nicotine replacement increases cessation rates over and above brief counseling + nicotine replacement alone. Finally, this study will provide valuable new information on TMI utilization patterns among homeless young people that will likely be useful to other researchers and clinicians who work with this population and are considering this mode of intervention delivery for health-related programming, as well as pave the way for the larger planned trial to evaluate the proposed cessation intervention.

## Data Availability

Once collected, deidentified data from this study will be available from the corresponding author on reasonable request 1 year after all aims of the project are completed. Requestors of data will be asked to complete a data-sharing agreement that provides for (1) a commitment to using the data only for research purposes and not to identify any individual participant; (2) a commitment to securing the data using appropriate computer technology; and (3) a commitment to destroying or returning the data after analyses are completed.
